# Supplementary material for: Mapping the evolution of fertility support policies in China: A content and instrumental analysis
Source: PLoS One. 2025 Oct 9;20(10):e0332137. doi: 10.1371/journal.pone.0332137 (PMC12510515; doi:10.1371/journal.pone.0332137)
Supplement: S1 Appendix — (ZIP) [file pone.0332137.s001.zip › S1 Appendix. 226 original policy documents/122-审计署关于提升社会保障审计监督效能的指导意见(FBM-CLI-4-5054796).doc]

审计署关于提升社会保障审计监督效能的指导意见

**发布部门：** [审计署](https://www.pkulaw.com/law/chl?Aggs.IssueDepartment=60501) [机构沿革](javascript:void(0))

**发布日期：**2021.07.26

**实施日期：**2021.07.26

**时效性：** [现行有效](https://www.pkulaw.com/law/chl?Aggs.TimelinessDic=01)

**效力级别：** [部门规范性文件](https://www.pkulaw.com/law/chl?Aggs.EffectivenessDic=XE0302)

**法规类别：** [审计综合规定](https://www.pkulaw.com/law/chl?Aggs.Category=03601)

审计署关于提升社会保障审计监督效能的指导意见

各省、自治区、直辖市和计划单列市、新疆生产建设兵团审计厅（局），署机关各单位、各派出审计局、各特派员办事处、各直属单位：
　　为深入贯彻习近平总书记关于审计工作的重要讲话和重要指示批示精神，落实党中央社会保障决策部署，提升社会保障审计（以下简称社保审计）监督效能，推动社会保障事业高质量发展，根据《“十四五”国家审计工作发展规划》，制定本意见。
　　一、总体要求
　　**（一）**指导思想。
　　坚持以习近平新时代中国特色社会主义思想为指导，深入贯彻党的十九大和十九届二中、三中、四中、五中全会精神，立足新发展阶段，贯彻新发展理念，构建新发展格局，以增进民生福祉、促进社会公平正义为目标，以保障资金运行“精准、安全、高效”为主线，把实现好、维护好、发展好最广大人民根本利益作为出发点和落脚点，更加关注困难群众的基本生活保障，更加关注民生政策的普惠性、基础性和兜底性，依法全面履行审计监督职责，一体推进揭示问题、规范管理、促进改革，促进健全覆盖全民、统筹城乡、公平统一、可持续的多层次社会保障体系，推进社会保障治理法治化、精细化，推动社会保障事业高质量发展、可持续发展。

　　**（二）**基本原则和主要目标。
　　牢牢把握审计机关首先是政治机关的定位，坚持党的全面领导，坚持以人民为中心，坚持依法审计、实事求是，坚持科技引领、改革创新，坚持系统观念，树立战略眼光，增强风险意识，推动党中央决策部署和各项改革任务贯彻落实。
　　--推动党中央社会保障有关决策部署落地生效。维护社会保障制度统一性、规范性，促进解决社会保障发展不平衡不充分问题和人民群众急难愁盼问题，保障改革发展成果更多更公平惠及全体人民。
　　--有效维护社会保障资金安全。守护好人民群众的每一分“养老钱”、“保命钱”和每一笔“救助款”、“慈善款”，及时揭示、防范和化解影响社会保障制度和资金可持续运行的重大风险隐患。
　　--促进不断提升社会保障治理效能。坚持问题导向、精准发力，做到应审尽审、凡审必严、严肃问责，形成常态化监督震慑，推动主管部门健全治理体系，健全社会保障权力运行制约机制，扣牢管理和控制责任链条，提高治理能力。
　　--有序推进社会保障领域审计全覆盖。健全社保审计工作组织领导制度，明确各类资金和项目的审计频率和覆盖周期，分级负责、突出重点，统筹审计资源，优化组织方式，消除监督盲区，推进纵向统筹和横向衔接，做到上下穿透、有机贯通，加快实现社保审计工作全国一盘棋。
　　--系统构建社会保障大数据审计工作模式。完善社保审计数据规划和采集机制，在署、省两级部署搭建社保审计大数据分析平台，开展常态化集中分析，充分运用大数据审计技术方法，不断提高社保审计能力、质量和效率。
　　二、聚焦主责主业，严守资金安全和风险底线

　　**（三）**进一步加大社会保障领域重大政策措施贯彻落实情况跟踪审计力度。
　　--就业优先政策落实方面。围绕更加充分更高质量就业目标，关注职业技能提升行动、创业带动就业、减税降费、普惠金融、稳岗扩岗、就业困难人员帮扶、新就业形态劳动者权益保障等保就业、保基本民生、保市场主体举措实施效果，推进落实高校毕业生、退役军人、农民工等重点群体就业保障政策，促进健全就业公共服务体系，提高就业资金使用效益。
　　--社会保险制度改革方面。围绕制度改革系统集成、协同高效目标，着力揭示制度整合不到位、转移接续不通畅等不衔接、不配套问题，推进基本养老保险全国统筹和基本医疗保险、失业保险、工伤保险省级统筹，促进发展完善多层次、多支柱养老保险体系，促进协同推进“三医联动”、药品耗材集中带量采购、门诊共济保障、重大疾病医疗保险和救助、医保支付方式改革等部署落实到位，促进社会保险制度公平和可持续发展。
　　--住房保障体系建设和改革推进方面。围绕让全体人民住有所居目标，聚焦城镇老旧小区改造、保障性租赁住房和共有产权住房建设、住房租赁市场发展等重大民生工程、重要政策措施推进情况，促进保障性安居工程、住房公积金、住宅专项维修资金等资金资产的公平善用，促进完善住房保障体系，提高保障性住房有效供给，推动城市更新建设，促进解决困难群众和大城市新市民、青年人等重点群体住房问题。
　　--社会救助、社会福利等社会保障兜底机制方面。围绕应保尽保、应助尽助、应享尽享目标，推动完善优化分层分类、城乡统筹的社会救助体系，健全退役军人保障制度，健全老年人关爱服务体系，完善帮扶残疾人、困境儿童等社会福利制度，促进将特殊困难群体基本生活保障到位，兜牢基本民生保障底线。
　　--社会保障公共服务和经办管理方面。围绕完善全国统一的社会保险公共服务平台目标，关注社会保险信息系统管理运行情况，推动数据整合和信息共享，深入推进社保经办数字化转型。促进健全社会保障对象精准认定机制，提高运用大数据主动发现困难群众的能力，加快社会保障“一卡通”管理，加强社会保险、住房、税务、民政、金融等信息的互联互通，提高社会保障公共服务的精准化、便捷化、智慧化水平。

　　**（四）**进一步加大对重点民生资金和项目审计力度。
　　--加强社会保险基金审计。全面掌握基金收入、支出、结余规模，摸清基本养老、基本医疗、失业、工伤等社会保险底数，以及职业年金、企业年金等补充养老、补充医疗保险情况，结合各项社会保险制度改革进程，揭示基金征缴、发放、管理和投资运营中的问题，维护基金安全和可持续运行。
　　--开展社会救助、社会福利和优抚安置资金审计。加大对最低生活保障、特困人员供养、医疗救助、残疾人补贴、优抚安置、彩票公益金等资金的审计，掌握政策覆盖面，摸清资金规模、保障对象和保障水平增长变化情况，重点关注资金申请、审核、分配、使用等环节存在的突出问题，查处套取骗取、挤占挪用、截留滞拨等违法违规问题，促进提高资金使用效益。
　　--积极开展应对人口老龄化、慈善捐赠等公共资金审计。审查普惠托育、养老服务、长期护理保险、殡葬、慈善捐赠等资金投入和使用绩效情况，摸清资金收支规模和结构情况，揭示资金管理使用和行业发展运营中存在的突出问题，促进发展普惠托育服务体系，构建居家社区机构相协调、医养康养相结合的养老服务体系，保障社会公益和慈善事业健康发展，落实积极应对人口老龄化国家战略。

　　**（五）**进一步加大对社会保障领域违纪违法问题和重大风险隐患的揭示力度。
　　--以零容忍态度严肃查处群众身边的微腐败和违纪违法问题。研究和把握社会保障领域欺诈骗保、与中介机构内外勾结非法牟利等违纪违法行为新手法、新动向，紧盯资金分配、项目审批、拆迁补偿、待遇发放、补贴申领、资格鉴定等重点环节，着力揭示骗取侵占、克扣截留、贪污受贿、失职渎职、优亲厚友等违纪违法问题。及时移送审计发现的违纪违法问题线索，协同有关部门和单位及时查处，推动对社会保障领域违纪违法行为早发现、早查处，坚决纠治损害群众切身利益的突出问题和不正之风，及时曝光重大典型案件情况，督促主管部门严肃查处就业、参保、安居工程等目标任务考核中的数据造假行为，及时补齐治理体系和治理能力短板。
　　--揭示影响社会保险基金可持续运行的重大风险。以社会保险基金预算执行和决算草案审计为抓手，分析社会保险基金收入、支出、结余变化趋势，检查各级财政对社会保险基金缺口分担、优先保障民生支出投入等保障机制落实情况，及时揭示一些地方社会保险基金“穿底”、划转国有资本充实社会保险基金不到位、自行增加待遇项目或提高待遇标准、城乡区域或群体之间保障待遇差异过大、基金投资运营损失等风险隐患，促进建立民生支出清单管理制度，完善社会保险基金支出预算管理，纠正和防范“过度保障”和“泛福利化”倾向，实现民生改善与经济发展良性循环。
　　--揭示影响公共安全和社会稳定的风险隐患。关注相关部门和地方突发公共事件预防、应急准备、监测和预警等情况，及时开展应对重大突发公共事件专项资金和捐赠款物审计，跟踪突发公共事件应对和困难群众基本生活保障情况，揭示应急物资储备和保障供应不足、困难群众应保未保等问题，推动健全自然灾害等重大突发公共事件应急处置和物资保障体系，促进完善社会保障应对突发重大风险的应急响应机制。
　　三、加强统筹谋划，加快推进社保审计工作全国一盘棋

　　**（六）**完善社保审计规划计划，高质量推进审计全覆盖。各级审计机关要摸清社保审计对象底数，聚焦重大战略、重大举措、重大项目、重大资金和重点人群保障，分析掌握政策实施和资金运行的周期性变化特征，建立本地区“十四五”社保审计工作发展规划，科学编制年度审计项目计划，做好与中长期审计项目库的有机衔接。上级审计机关要着力聚合审计目标，抓好统筹管理和监督检查，避免重复审计和相互割裂，推动计划落实落地。社保审计部门要根据社会保障资金的重要性、规模和管理分配权限等因素，确定重点审计对象，合理安排社保审计项目的时机、节奏、重点和方式，通过专项审计、政策措施落实情况跟踪审计、预算执行与决算草案审计等相结合，对涉及的重点部门和单位进行重点监督，对医保、养老等群众关切、问题多发的重点资金进行常态化审计监督。

　　**（七）**健全分级负责、协同联动的组织实施机制。审计署主要通过规划计划、工作调度、请示报告、监督考核、督查督办、规范指引、项目评优、总结培训等方式，做好对地方审计机关社保审计工作的组织领导和业务指导；每年常态化组织开展社会保障领域重大政策措施落实情况跟踪审计，适时统一组织对涉及全局的重点专项资金、重大民生项目、重大突发公共事件开展专项审计。地方审计机关按照审计管理权限抓好以地方财政事权和支出责任为主、管辖范围内的社会保障资金和项目审计。各级审计机关要坚决贯彻落实署党组关于社保审计工作以地方审计机关为主实施的要求，加强协调联动，组织做好不同审计项目中涉及社会保障相关内容的衔接，形成覆盖中央和地方，贯穿社会保障资金筹集、分配、使用、管理及投资运营等各环节的全链条穿透式审计。要充分利用内部审计资源，发挥内部审计作用，根据审计项目实施需要，可以按规定向社会购买审计服务。

　　**（八）**坚持严查实报，完善审计结果报告和共享机制。各级审计机关社保审计项目综合情况报告、审计重要信息等审计结果应向上级审计机关报告。社保审计项目综合情况报告要反映审计对象总体情况、存在的主要问题，从体制性障碍、机制性缺陷、制度性漏洞等方面深入分析原因，提出可操作的、有针对性的审计建议，便于主管部门、被审计单位研究落实。对于上级审计机关统一组织的社保审计项目，必须严格执行重要审计情况、重大违纪违法问题及处理情况等重大事项请示报告制度，并及时向上级审计机关报告本地区社保审计项目工作总结。上级审计机关要加强对社保审计报告、审计信息等审计结果的汇总分析和综合利用，审计机关之间要建立顺畅高效的沟通协调机制，在符合保密要求、遵循规定程序的前提下实现信息共享、一果多用。

　　**（九）**加强整改督促，切实推动解决问题和标本兼治。审计中要坚持边审边促、边审边改、边审边建，及时开展对审计查出问题整改情况的督促检查，推动被审计单位压实整改主体责任，强化主管部门监督管理责任，确保审计查出问题整改落实到位。对审计查出的问题，要明确整改责任主体，科学合理、分类施策提出整改要求。对决策层面问题重在治本，着力促进主管部门健全完善政策、制度；对执行层面问题坚持标本兼治，既要监督问责，又要促进完善制度，形成长效机制，实现源头治理。完善情况通报、资料提供、线索移送等相关工作机制，做好与纪检监察、巡视巡察、人大监督、司法监督、党委和政府督查等有机贯通和协调配合，推动审计整改取得实效。深化审计结果和整改情况公开，推动系统性解决问题。
　　四、以大数据审计为支撑，提高社保审计能力、质量和效率

　　**（十）**完善社保审计数据采集和定期报送机制。审计署加强社保审计数据标准、采集和应用的顶层设计，建立常态化数据采集机制。地方审计机关按照分工负责辖区内的数据采集工作，定期采集社会保障相关财务业务数据并逐级上报，保证数据的完整性、连续性、时效性。以金审工程三期项目建设为依托，在署、省两级集中部署数据归集和分析环境，实现社会保障数据跨层级、跨地域、跨系统集中分析；在省级建立特派办与地方审计机关之间的数据分析及结果共享机制，提高数据运用效率。

　　**（十一）**扎实推进社会保障大数据审计。各级审计机关要在社保审计工作中积极推广“总体分析、发现疑点、分散核实、系统研究”的数字化审计模式，综合运用关联比对、多维分析、聚类分类等方法，对社会保障相关数据开展常态化集中分析。突出对违纪违法违规问题的分析核查，根据问题特征研究审计思路、构建数据分析模型，提高发现问题能力；围绕改革发展方向和重点关注领域开展专题分析，揭示苗头性、倾向性问题，提高风险感知能力；从宏观层面进行结构和趋势分析，对社会保障政策运行总体情况和存在的问题进行系统评价，提高研判宏观趋势能力；将经过审计实践验证的技术方法数据化、标准化、体系化，开发建设社会保障大数据审计模型库，探索建立社保审计实时监督系统和联网审计模式，提高智能审计能力。
　　五、加强组织领导，为提升社保审计效能提供有力保障

　　**（十二）**提高政治站位，切实加强组织领导。各级审计机关和全体审计人员要深刻认识和把握社会保障“民生保障安全网、收入分配调节器、经济运行减震器”的重要地位和作用，坚定不移把讲政治贯穿审计工作始终，树立正确的民生审计政绩观，提高政治判断力、政治领悟力、政治执行力，将人民群众获得感、安全感、幸福感提升作为衡量社保审计成效的重要考量，善于用政治眼光观察和分析审计发现的经济社会问题，以改革的办法和创新的思维解决发展中的问题，确保经得起历史和人民检验。地方各级审计机关要扛起社保审计项目实施主体责任，强化规划计划引领和约束，统筹整合审计资源，加大审计力量投入，抓好各项计划任务的落实落地，切实履行本地区社保审计监督职责，真正做到守土有责、守土担责、守土尽责。

　　**（十三）**走实群众路线，深入开展研究型审计。社保审计工作要全面贯彻党的群众路线，遵循社会保障与经济发展之间“水涨船高”的客观规律，吃透党中央有关决策部署的政治意图、战略规划和实践要求，系统把握我国社会保障事业的历史沿革、发展现状和改革方向，深入研究社会保障资金、政策、项目和业务特点及问题特征，吸收借鉴专家学者等各方面的观点意见，提高精准揭示和有力有效解决问题的能力。开展全国性、区域性和新领域的审计项目前，要通过试点审计把握总体情况、明确审计思路、找准审计重点，探索因地制宜、分片分主题的协同组织方式，加强统筹谋划，做到科学布局、有的放矢。审计工作方案和实施方案要坚持问题导向、突出重点，明确抽查资金比例和抽查面，增强方案的操作性、指导性。

　　**（十四）**严格质量控制，不断提高专业水平。审计实施中要顺着资金流向深入到基层、延伸到保障对象，核查要见人、见账、见物，盯紧盯住政策落实的“最后一公里”、资金运行最末端，做到以点带面、审深审透。牢固树立“有问题没发现是失职、发现问题不报告是渎职”的意识，敢于较真碰硬，查真相、说真话、报实情。严守政治纪律、工作纪律、廉政纪律和保密纪律，自觉运用法治思维分析和处理问题，做到程序合规、边界清晰，确保审计权力在法治轨道上运行。进一步健全社保审计制度规范体系，优化审计运行机制和工作流程，开展分层次、分类型的专业培训和专题研究，加强社保审计专业化队伍培养和专业能力建设，为社保审计高质量发展夯实基础。

审计署
　　2021年7月26日

© 北大法宝：（[www.pkulaw.com](https://www.pkulaw.com/)) 专业提供法律信息、法学知识和法律软件领域各类解决方案。北大法宝为您提供丰富的参考资料，正式引用法规条文时请与标准文本核对。
欢迎查看所有[产品和服务](https://www.pkulaw.com/helps/2.html)。法宝快讯：[如何快速找到您需要的检索结果？](https://www.pkulaw.com/helps/70.html)[法宝](https://www.pkulaw.com/helps/69.html)V6有何新特色？

扫描二维码阅读原文

**原文链接：**<https://www.pkulaw.com/chl/a8f66eca7ff9f47abdfb.html>
